# Functional phenomics and genetics of the root economics space in winter wheat using high‐throughput phenotyping of respiration and architecture

**DOI:** 10.1111/nph.17329

**Published:** 2021-04-26

**Authors:** Haichao Guo, Habtamu Ayalew, Anand Seethepalli, Kundan Dhakal, Marcus Griffiths, Xue‐Feng Ma, Larry M. York

**Affiliations:** ^1^ Noble Research Institute LLC 2510 Sam Noble Parkway Ardmore OK 73401 USA

**Keywords:** genome‐wide association studies (GWAS), multi‐trait, root architecture, root respiration, winter wheat

## Abstract

- The root economics space is a useful framework for plant ecology but is rarely considered for crop ecophysiology. In order to understand root trait integration in winter wheat, we combined functional phenomics with trait economic theory, utilizing genetic variation, high‐throughput phenotyping, and multivariate analyses.
- We phenotyped a diversity panel of 276 genotypes for root respiration and architectural traits using a novel high‐throughput method for CO_2_ flux and the open‐source software rhizovision explorer to analyze scanned images.
- We uncovered substantial variation in specific root respiration (SRR) and specific root length (SRL), which were primary indicators of root metabolic and structural costs. Multiple linear regression analysis indicated that lateral root tips had the greatest SRR, and the residuals from this model were used as a new trait. Specific root respiration was negatively correlated with plant mass. Network analysis, using a Gaussian graphical model, identified root weight, SRL, diameter, and SRR as hub traits. Univariate and multivariate genetic analyses identified genetic regions associated with SRR, SRL, and root branching frequency, and proposed gene candidates.
- Combining functional phenomics and root economics is a promising approach to improving our understanding of crop ecophysiology. We identified root traits and genomic regions that could be harnessed to breed more efficient crops for sustainable agroecosystems.

## Abstract 2

See also the Commentary on this article by Li *et al*., **232**: 5–7.

## Introduction

Functional phenomics is an emerging transdisciplinary field that integrates physiology, high‐throughput phenotyping, and computational biology in order to fill gaps in our knowledge of various aspects of plant functioning (York, 2019). High‐throughput phenotyping allows for large‐scale data collection on plant form and function, and it is often used for studies of genetics within a species. Phenomics focuses on understanding variation in plant phenotypes, but it often lacks analysis of the relationship between phenotypes and function, even if quantitative genetics are employed. The use of functional phenomics is therefore required, in which statistical associations within high‐dimensional phenomics datasets can be analysed to infer how traits influence one another, and how they influence the physiological processes that are important for crop growth. In particular, root phenomics data and conceptual frameworks are lacking, resulting in a poor understanding of their interactions and integration, as described in York *et al*. (2013). The trait economics spectrum is a conceptual framework from ecology that could help explore trait integration in crops. In this context, economics refers to the balance among traits for resource acquisition and utilization, with an explicit treatment of the tradeoffs between pairs of traits (Reich, 2014). For example, in a controlled study of 74 plant species, a root economics spectrum was found in which root respiration was correlated with percent nitrogen, root length per unit mass, and the decomposition rate of dried roots in soil (Roumet *et al.,* 2016). Recently, a two‐dimensional root economics space was proposed, formed by one gradient which represents whether or not to cooperate with fungal partners, and a second confirming the conventional fast–slow ‘conservation’ gradient (Bergmann *et al.,* 2020). Interestingly, the first dimension was partially driven by specific root length, a proxy for structural cost, and the second axis by root nitrogen content, a proxy for specific root respiration and metabolic cost. Therefore, the root economics space is a useful framework for understanding carbon use efficiency in crop roots.

Roots constitute the interface between plants and soil, and a key function of roots is the extraction of the nutrients and water required for plant productivity (Smith & De Smet, 2012; Meister *et al.,* 2014). However, there is a complex relationship between investment in the root system and plant productivity, because there is a cost associated with roots. The fraction of newly fixed carbon from photosynthesis allocated to roots can exceed 50%, and this proportion significantly increases under edaphic stress (Lambers *et al.,* 1996; Rachmilevitch *et al.,* 2015). Root system carbon costs can be classified as structural costs (the physical structure of the roots and growth respiration) and maintenance costs (upkeep respiration and exudation) (Mooney, 1972; Kong & Fridley, 2019; Sun *et al.,* 2021). For example, in wheat seedlings, 30% of net photosynthesis was associated with root structural and maintenance costs (Sawada, 1970). Optimization of the structure and metabolism of the root system would therefore have a significant impact on plant carbon use efficiency.

Specific root length is a measure of the carbon expenditure required to increase root length, and is often given in units of m g^−1^. Specific root respiration standardizes respiration based on root length or mass, typically with units of nmol CO_2_ s^−1^ cm^−1^ or mg^−1^, respectively. Specific root length was found to exhibit variation among a set of barley and wheat lines, but the genetic contribution was not explicitly considered (Løes & Gahoonia, 2004), and it was used for analysis of quantitative trait loci (QTLs) in common bean (Ochoa *et al.,* 2006). Across the plant kingdom, as much as 52% of current photosynthates may be respired by plant roots during a single day, depending on the species and environmental conditions (Lambers *et al.,* 1996). Plant respiration uses substrates from photosynthesis to produce carbon skeletons and usable energy, and in chemical reduction processes required for development (Amthor, 2000); the process of respiration in plants entails the consumption of oxygen and the release of carbon dioxide. A multicomponent framework has been suggested, in which respiration can be divided into three parts: a growth fraction – the biosynthesis of new structural biomass and exudates; a maintenance fraction – the translocation of photosynthates from sources to sinks, and cellular ion‐gradient maintenance; and an ion‐uptake fraction, including the uptake of ions, assimilation of nitrogen and sulphur, and protein turnover (McCree, 1970; Thornley, 1970; Johnson, 1983; Poorter *et al.,* 1991; Amthor, 2000). As up to 60% of assimilated carbon is lost through respiration, strategies for the minimization of unnecessary respiratory activity could lead to substantial gains in crop productivity by enhancing plant carbon use efficiency (Amthor *et al.,* 2019; Weber & Bar‐Even, 2019; Roell & Zurbriggen, 2020).

Variation in root respiration rates among crop species occurs due to differences in root tissue density, anatomy, activity, chemistry, and structure (Ben‐Noah & Friedman, 2018). Studies have shown that the reduction of root respiration through anatomical changes, such as root cortical senescence in barley (*Hordeum vulgare*) and wheat (*Triticum aestivum*) (Schneider *et al.,* 2017), or reduction in root secondary growth, such as that observed in common bean (*Phaseolus vulgaris*), (Strock *et al.,* 2018) permit greater plant growth by improving phosphorus capture from low‐phosphorus soils. Strategies that have been proposed and/or used to reduce root respiratory carbon cost for the improvement of plant performance include making ion transport more efficient (Amthor *et al.,* 2019), manipulation of the genes or enzymes involved in carbon metabolism in plant roots (Dorion *et al.,* 2017; Florez‐Sarasa *et al.,* 2020), and the use of arbuscular mycorrhizal symbiosis to reduce root respiratory rate as well as increasing photosynthesis (Romero‐Munar *et al.,* 2017). Root respiration that is not accounted for by necessary plant functions can be referred to as ‘luxury’ respiration.

Understanding the genetic bases of specific root length and respiration, among other traits, as well as their relationship to plant performance, is of key importance for breeding more productive and resilient crop varieties to adapt to climate change. However, these traits have rarely been considered as a unit of phenotype for breeding or genetic mapping. Genome‐wide association studies (GWAS) for respiratory traits typically require many hundreds of plant variants, as well as the measurement of respiratory traits at the same time of day and at the same developmental stage (Scafaro *et al.,* 2017). Infrared gas analyzers for portable leaf photosynthesis or O_2_‐electrode techniques are commonly used to measure rates of root respiration (Poorter *et al.,* 1991; Strock *et al.,* 2018), but most of those protocols are low‐throughput, and require costly instruments that are less flexible in terms of outputting data in convenient formats. Addressing the need for rapid, cost‐effective, large‐scale root respiratory screening will require the development of both high‐throughput root respiration measurement and data analysis capabilities, the combination of which will greatly strengthen functional phenomics by increasing statistical power and enabling genetic mapping (York, 2019).

Wheat, a member of the grass family, is an important cereal that is grown globally. Winter wheat in the Southern Great Plains of the United States is often grown as a dual‐purpose crop for forage and grain production (Maulana *et al.,* 2019). Yield, protein content (Rajaram, 2001), disease resistance (Ellis *et al.,* 2014), and heat resistance (Maulana *et al.,* 2018) are major targets for modern wheat breeding and genetic improvement. Significant marker–trait associations for aboveground traits, such as yield and its components (Sukumaran *et al.,* 2018) and nitrogen use efficiency (Cormier *et al.,* 2016; Hawkesford & Griffiths, 2019), have been reported across the wheat genome. Indeed, a considerable number of QTLs associated with wheat root traits have been identified on nearly all chromosomes in variable environments (Hamada *et al.,* 2012; Bai *et al.,* 2013; Atkinson *et al.,* 2015; Maccaferri *et al.,* 2016; Xie *et al.,* 2017; Beyer *et al.,* 2019; Soriano & Alvaro, 2019). However, understanding of the genetic and functional bases of root traits still lags behind that of aboveground traits, and genetic variation of root structural and metabolic traits remains underexplored. Accordingly, this study was conducted with the following objectives: to develop a high‐throughput phenotyping platform that integrates a hydroponics growth system, infrared gas analyzers, custom gas chambers, a bead bath, flatbed scanners, analytical scales, and an R script for measuring specific root respiration, specific root length, and other root traits; to validate the platform using winter wheat to uncover heritable variation of root respiration and architectural traits; to employ functional phenomics to identify relationships among traits and tissue‐type dependencies; and to identify associated QTLs/genes that drive root respiration and other root traits by performing GWASs.

## Materials and Methods

### Plant materials

The plant materials were selected from the hard winter wheat association mapping panel (HWWAMP) of the Triticeae Coordinated Agricultural Project (T‐CAP). A total of 276 hard winter wheat (*Triticum aestivum* L.) cultivars and breeding lines were selected from the panel, which covers a broad range of selection and breeding history in the Great Plains of the USA.

### Experimental design

The 276 wheat lines were grown as two replicates in a single growth chamber (552 plants), and the entire procedure was repeated twice, for a total of four replicates and 1104 plants. Each replicate was treated as a block for an overall experiment with a randomized complete block design. The seedlings were transplanted into the hydroponic grow boxes on 19 June and 4 October 2019. The details of the germination, growth, and sampling procedures are given in the paragraphs that follow.

### Growth conditions

**Fig. 1:**
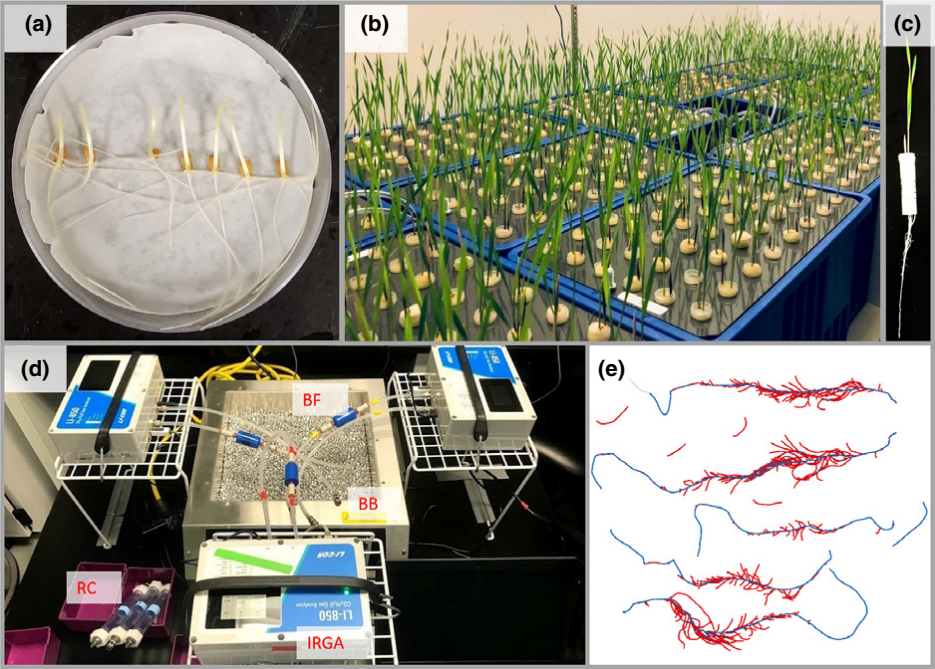
Platform for phenotyping root respiration and other root traits of winter wheat seedlings. (a) Seeds were surface sterilized and pre‐germinated in Petri dishes. (b) Seedlings were grown in aerated hydroponics for 10 d. (c) Shoot and roots of seedlings 10 d after transplanting. (d) Root respiration was measured in an airtight chamber using an LI‐850 analyzer with temperature control and a bead bath. (e) Axial roots (blue) are distinguished from lateral roots (red) in a scanned image using rhizovision explorer. BB, bead bath; BF, Balston Filter; IRGA, infrared gas analyzer; RC, root chamber.

Seeds were surface‐sterilized in 0.5% sodium hypochlorite (NaOCl) for 10 min, rinsed three times using deionized (DI) water, and pre‐germinated in Petri dishes with filter paper which were placed in darkness at 25°C for 3 d. Uniformly germinated seedlings were selected (Fig. 1a), wrapped around the root–shoot junction with L800‐D Identi‐Plugs foam (Jaece Industries, North Tonawanda, NY, USA), plugged in a 15 ml Falcon conical centrifuge tube (Corning Inc., Corning, NY, USA) with the bottoms cut away from the 6 ml mark, and transplanted into a hole cut into the lid of the growth system (Fig. 1b). A unique barcode label was affixed to each tube for sample identification. The hydroponics growth system consisted of a polypropylene divider box (inside dimensions: length 38.10 cm, width 22.86 cm, height 20.32 cm, volume 17.7 l) and a custom lid made from a PVC panel cut to fit into the top of the box (4.5 mm thick × 250 mm wide × 392 mm long, with the corners cut off to accommodate the box’s rounded corners). Forty‐eight holes of 18 mm diameter were drilled into the lid using a hole saw, leaving equal spacing between holes. Twelve growth boxes were placed in a Conviron E‐15 growth chamber (Conviron, Winnipeg, Canada) with a 16 h : 8 h, light : dark photoperiod at 25 : 20°C, with a flux density at canopy level of *c*. 400 µmol m^−2^ s^−1^. Each box was filled to the bottom of the lid with a nutrient solution containing 125 µM KH_2_PO_4_, 1125 µM KNO_3_, 500 µM CaCl_2_, 250 µM MgSO_4_, 11.5 µM H_3_BO_3_, 1.75 µM ZnSO_4_·7H_2_O, 2.25 µM MnCl_2_·4H_2_O, 0.08 µM CuSO_4_·5H_2_O, 0.03 µM (NH_4_)_6_Mo_7_O_24_·4H_2_O, and 19.25 µM Fe(III)‐EDTA (C_10_H_12_N_2_NaFeO_8_). The nutrient solution was continuously aerated with an air pump attached to airstones submerged in each growth box, and the solution pH was maintained between 5.9 and 6.1 by the addition of KOH or HCl throughout the experiment.

### High‐throughput root respiration measurements

Ten days after transplanting (Fig. 1c), plants were removed from the nutrient solution. Roots were immediately excised from shoots, blotted using tissue paper to remove excess water, and placed in a 19 ml custom chamber connected to an LI‐850 CO_2_/H_2_O Analyzer (Li‐Cor Inc., Lincoln, NE, USA) (Fig. 1d). The custom chamber was made from a 12.7 mm (sold as ½ inch) internal diameter clear polyvinyl chloride (PVC) pipe nipple (United States Plastic Corp., Lima, OH, USA), which was 152.4 mm in length with threaded ends. Holes were drilled into 12.7 mm female national pipe thread (FNPT) nylon threaded caps (United States Plastic Corp.) in order to accommodate the insertion of quick‐connect bulkhead male or female fittings (Li‐Cor) with rubber grommets to create a seal. A Balston filter (Li‐Cor) was inserted between the chamber and the analyzer to filter air. The chamber was buried in a Lab Armor 20 l bead bath (Lab Armor LLC, Irving, TX, USA) filled with Lab Armor metallic beads, with the temperature set at 28°C. Beads were preferred to water in order to prevent contamination of the system with water. The chamber CO_2_ concentration was continuously recorded using li‐850 windows software v.1.0.2 for 90 s at a rate of one reading s^–1^. A USB barcode scanner (TaoTronics, Fremont, CA, USA) was connected to each laptop to acquire and save the datafile with the appropriate sample name, encoded by the barcode affixed to the cut tube described in the section on growth conditions. Three infrared gas analyzers were used to allow simultaneous respiration measurements in parallel to increase throughput, which reached 25 samples per person h^−1^, with a team of eight.

In order to calculate the total respiration rate of a root sample from the individual text files containing the time series molar fraction of CO_2_, an R (R Core Team, 2018) script was developed in order to load each text file from a directory, conduct a series of computations, and output the total respiration rate. Total respiration rate (CO_2_ flux) was calculated using Eqn 1.

(Eqn 1) $$F=\frac{\mathrm{PV}}{\mathrm{RT}}\frac{dC}{dt}$$

where *F* is the CO_2_ flux in nmol s^−1^, P is the pressure in the chamber in kPa, V is the corrected chamber volume in ml, *R* is the ideal gas law constant in l kPa K^−1^ mol^−1^, *T* is air temperature in K, and d*C*/d*t* is the change in CO_2_ concentration on a molar basis with time (µmol mol^−1^ s^−1^). Chamber volume (*V*) was determined by subtracting the total root volume, estimated using rhizovision explorer, from the chamber volume.

For root respiration analysis, the dead band (length of initial time to be ignored) was set at 20 s. To estimate d*C*/d*t*, the slope was used from a linear regression fit to the water‐corrected CO_2_ concentration provided by the LI‐850 analyzer over the corresponding observation time (20–90 s) using the *lm* function in R (R Core Team, 2018). The protocol for the root respiration measurements and the R script for calculating total flux from a directory of text files are available at https://doi.org/10.5281/zenodo.4247873 (Guo *et al.,* 2020a).

After the root respiration measurements, roots were stored at 4°C and scanned within 1 wk. Roots from each plant were spread out in 5 mm of water in transparent acrylic trays and imaged with an Expression 12000XL flatbed scanner equipped with a transparency unit (Epson America, Los Alamitos, CA, USA) at a resolution of 600 dpi. Given the small size of the root systems, which were already clean because they were grown hydroponically, scanning throughput was 15 samples per person h^−1^. Images were analyzed using rhizovision explorer v.2.0.2 (Seethepalli & York, 2020) and algorithms described by Seethepalli *et al.,* (2020), with the options for image thresholding level, filtering of noisy components, and threshold for root pruning being set at 205 pixel intensity, 0.2 mm^2^, and 1 pixel, respectively. A root diameter threshold of 0.3 mm was used to distinguish axial roots from lateral roots (Fig. 1e).

The root traits extracted using rhizovision explorer in this study are as follows: number of root tips (Tip), number of branching points (BP), branching frequency (BF), total root length (TRL), axial root length (ARL), lateral root length (LRL), average diameter (AvgD), total root volume (TRV), axial root volume (ARV), lateral root volume (LRV), total root surface area (TSA), axial root surface area (ASA), and lateral root surface area (LSA). Branching frequency is determined by the software by dividing the number of branching points by total root length. When root scanning was completed, roots and shoots were dried at 60°C for 3 d before dry weight determination, with a throughput of 70 samples per person h^−1^. The oven‐dried root mass and root length quantified using rhizovision explorer were used to calculate the specific root respiration (SRR) per unit of root dry mass (SRR___M; nmol g^−1^ s^−1^) and the specific root respiration per unit of root length (SRR_L; nmol m^−1^ s^−1^), respectively.

Root mass fraction (RMF) was found by calculating the root dry weight as a proportion of the total plant dry weight. Specific root length (SRL) was calculated by dividing root length by the corresponding root dry weight. Lateral : axial root length ratio was calculated by dividing lateral root length by the corresponding axial root length, based on the diameter threshold provided during image analysis, and lateral : axial root volume ratio was calculated by dividing lateral root volume by the corresponding axial root volume. Branching density (BD) was calculated by dividing root tips by axial root length. Root tissue density (RTD) was calculated by dividing root dry weight by root volume, which brought the total number of traits reported in this study to 25.

Broad‐sense heritability (*H* ^2^) of each trait was calculated based on the equation described by Falconer & Mackay (1996):

$$H^{2}=\frac{\sigma_{g}^{2}}{\sigma_{g}^{2}+\frac{\sigma_{e}^{2}}{r}}$$

The variables $\sigma_{g}^{2}$, $\sigma_{e}^{2}$ and r represent the variance of the genotype effect, variance of the local environment effect, and the number of replicates (blocks), respectively. The variances were obtained by fitting to a mixed model including genotype as a random effect and block as a fixed effect using the lme4 package (Bates *et al.,* 2014).

Principal component analysis (PCA) and visualization of outputs were performed on the trait means of the 25 traits using the base function ‘prcomp’ and the R package factoextra (Kassambara & Mundt, 2017). The first ten principal component scores were extracted for clustering and PC‐based GWAS analysis (PC‐GWAS).

### Network analysis

Due to the high correlation between variables and singularities, root volume, surface area related traits, and lateral : axial root length ratio were dropped for network analysis. To assess the relationships among the remaining 17 traits, pairwise Pearson’s correlation coefficients (*r*) of the traits were estimated to construct a Gaussian graphical model (GGM) for network analysis. Network analysis with a Gaussian graphical model is a more holistic way to capture causality and precursor/product relationships in complex trait networks relative to standard correlation analyses. A GGM provides conditional dependence between two variables after removing the effects of all other variables to avoid spurious correlations (Krumsiek *et al.,* 2011; Carlson *et al.,* 2019). The network analysis and visualization of trait relationships were carried out using the R package qgraph (Epskamp *et al.,* 2012). Outdegree is the number of connections that a trait node has to other trait nodes. Betweenness centrality quantifies the number of times a trait node acts as a bridge along the shortest path between two other trait nodes.

### Multiple linear regression analysis

Multiple linear regression analysis was employed to determine how total respiration can be partitioned into the contributions from root tissue types. For this analysis, root volume was considered rather than mass because it could be derived from the image data for each root class without requiring physical dissection of the root system to acquire mass measurements. The total axial root volume, lateral root volume (minus the tip volume), and lateral root tip volume were used as the dependent variables, while the total root respiration was the independent variable. The number of lateral root tips was estimated by subtracting four from the number of root tips supplied by rhizovision explorer, assuming that the typical wheat seedling had four seminal roots, based on the counting of seminal roots in a limited subset. The average number of total tips was nearly 400, so this correction had minor effects. This number of lateral roots was multiplied by 0.01 mm^3^ in order to assign a small volume to the lateral root tips, which were assumed to be more active based on previous research (Ben‐Noah & Friedman, 2018). Lateral root axis volume was determined by subtracting lateral root tip volume from the total lateral root volume. Based on visual evaluation of feature images in rhizovision explorer, total lateral root volume and total axial root volume were assumed as the volumes of the diameter ranges ≤ 0.3 mm or > 0.3 mm, respectively. The ‘stepAIC’ function implemented in R package mass (Ripley *et al.,* 2020) was used for the stepwise regression, and it revealed this full model to be the most parsimonious, so residuals of this model were used as an additional trait (SRR_R) for subsequent analysis. SRR_R is the respiration that is not accounted for after considering root system architecture and root tissue dependency.

### Single nucleotide polymorphism (SNP) genotyping

High‐density SNP markers from the wheat 90K SNP genotyping array were obtained from genotype experiment TCAP90K_HWWAMP of The Triticeae Toolbox database (https://triticeaetoolbox.org/wheat/). Data constituting 21 555 SNPs were filtered to exclude markers with missing data greater than 50% and minor allele frequency < 5%, resulting in 16 058 makers that were used in the association analysis. The map positions for the SNP markers used in this study were based on the consensus map developed using a combination of eight mapping populations (Wang *et al.,* 2014).

### Genome‐wide association analysis

Three genome‐wide association analysis approaches were employed to identify genomic regions associated with various root traits. The linear mixed model (LMM) in gemma (Zhou & Stephens, 2012; Zhou & Stephens, 2014) was used to test for association between SNPs and traits. The population relatedness matrix was estimated using the centered relatedness algorithm within gemma, and was chosen as a covariate in the model to control population structure. A Wald test was performed to determine *P*‐values.

Single‐trait (Univariate) association testing was run for each of the 25 traits using mean phenotypic values, and PC‐GWAS was conducted using each of the first 10 PCs. Multi‐trait (multivariate) GWAS was carried out to increase the power of the association tests and to detect polymorphisms with potentially pleiotropic effects of trait‐associated loci using the multivariate linear mixed effect modeling capabilities of gemma. The 25 traits were grouped into six multi‐trait combinations based on their genetic correlations or their structural and functional relationships (McCormack *et al.,* 2017; Ben‐Noah & Friedman, 2018). Root dry weight and shoot dry weight were combined to form a biomass‐related multi‐trait set (biomass). Total root respiration, root dry weight, root mass fraction, number of root tips, axial root length, and branching density were combined to form a root‐respiration‐related multi‐trait set (root respiration) because these traits had functional relationships based on network analysis and provide a broader picture of root respiration. Axial root length, lateral root length, axial root volume, lateral root volume, axial root surface area, and lateral root surface area were combined to form a root‐morphology‐related multi‐trait set (morphology). Branching point, branching frequency, and branching density were combined to form a root‐topology‐related multi‐trait set (topology). Specific root length, root tissue density, and average root diameter were combined to form a root‐construction‐related multi‐trait set (construction). Root mass fraction, lateral : axial root length ratio, and lateral : axial root volume ratio were combined to form an allocation‐related multi‐trait set (allocation). Multi‐trait association was conducted with gemma using the multivariate version of the same model used for single‐trait associations.

Outputs from gemma were used to generate Manhattan and quantile–quantile (QQ) plots using the R package qqman (Turner, 2018). As mentioned in many wheat studies (Maulana *et al.,* 2018; Beyer *et al.,* 2019), determining a significance cutoff threshold is one of the biggest challenges for GWAS. Significant QTLs were initially tested based on a false discovery rate of 0.05 following a stepwise procedure, which is very stringent (Müller *et al.,* 2011). So, the negative logarithm of the raw *P*‐value (–log_10_P) ≥ 3.5 was used for detecting SNPs that are significantly associated with these complex quantitative traits, consistent with the process outlined by Maulana *et al*. (2018).

### Identification of candidate genes

The sequences of significant markers associated with phenotypic traits were downloaded from the Triticeae Toolbox database (Wang *et al.,* 2014), and were blast searched against the wheat genome in phytozome v.2.2 to identify candidate genes located ± 250 kb proximal to each identified marker. The ± 250 kb window was selected based on linkage disequilibrium analysis of this wheat panel (Ayana *et al.,* 2018; Maulana *et al.,* 2018), which reflects a relatively small interval. Candidate genes of interest were selected based on the criteria of close proximity to the SNP and possible involvement in the regulation of root development (based on a literature review).

## Results

### Variations of root respiratory and architectural traits

**Fig. 2:**
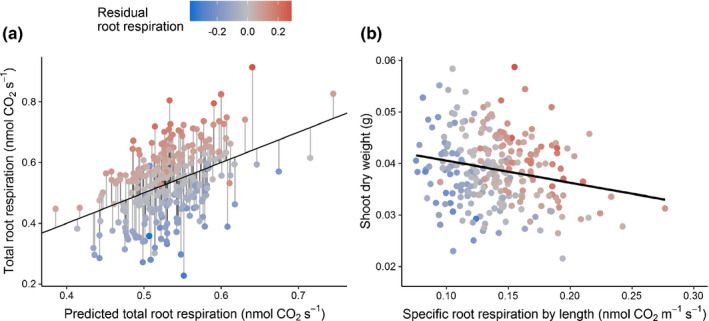
(a) The relationship between predicted total root respiration and total root respiration in winter wheat, and deviations from the relationship results in a new trait specific root respiration residual (SRR_R). (b) Regression between specific root respiration by length and shoot dry weight.

**Fig. 3:**
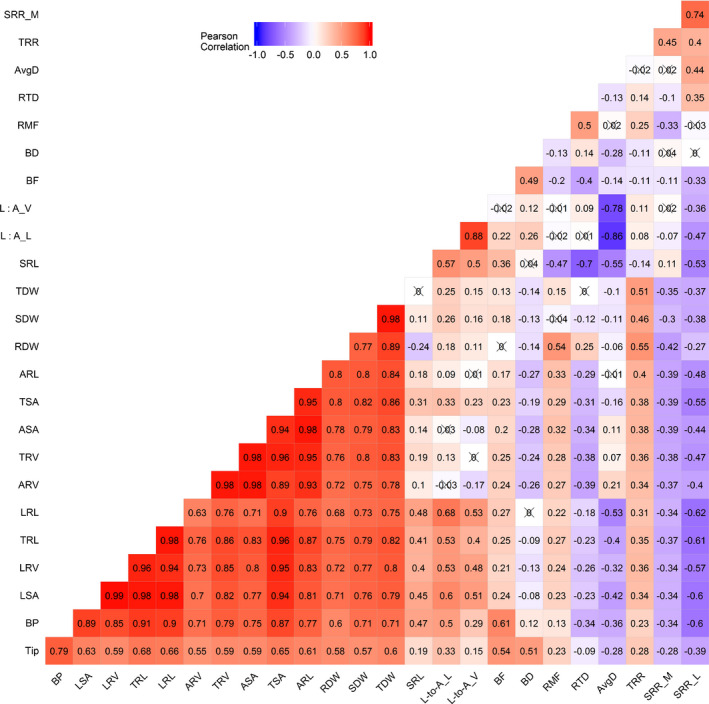
Pairwise Pearson correlation for selected traits of the Triticeae Coordinated Agricultural Project (T‐CAP) winter wheat seedlings. The numbers represent the correlation values. The cross symbol (×) means that the correlation value is not significant at *P* < 0.05. Bright red to bright blue indicates highly positive to highly negative correlations. Traits were measured in winter wheat. ARL, axial root length; ARV, axial root volume; ASA, axial root surface area; AvgD, average root diameter; BD, branching density; BF, branching frequency; BP, number of branch points; LRL, lateral root length; LRV, lateral root volume; LSA, lateral root surface area; L : A_L, lateral : axial root length ratio; L : A_V, lateral : axial root volume ratio; RDW, root dry weight; RMF, root mass fraction; RTD, root tissue density; SDW, shoot dry weight; SRL, specific root length; SRR, specific root respiration; SRR_L, SRR per root length; SRR_M, SRR per root mass; SRR_R, SRR residual; TDW, total dry weight; Tip, number of root tips; TRL, total root length; TRR, total root respiration; TRV, total root volume; TSA, total root surface area.

Shoot dry weight (SDW), root dry weight (RDW), total dry weight (TDW), total root respiration (TRR), SRL, lateral : axial root length ratio (L : A_L), ASA, lateral : axial root volume ratio (L : A_V), PC2, PC3, PC4 and PC7 exhibited normal distribution. Near normal distributions were observed for other root traits (Supporting Information Fig. S1). The root traits with > 5‐fold variation between maximum and minimum values in the wheat population were SRR_L, TRL, LRL, LRV, LSA and BP. 3.2‐fold and 2.2‐fold variations were observed in SRR_M and SRL, respectively, in the wheat population. Broad‐sense heritabilities ranged from 0.25 to 0.57 for the 25 traits (Table 1). The respiration residual, SRR_R, of a multiple regression fit (Fig. 2a) that accounts for respiration not explained by root system architecture, had a heritability of 0.44. The maximum heritability was observed for SDW (0.57). The root traits with heritabilities greater than 0.50 were SRL, BP and AvgD. Many strong correlations were observed among traits. Total root respiration had correlation values > 0.50 with RDW and TDW. Interestingly, specific root respiratory traits (SRR_L and SRR_M) had significant negative correlations with shoot, root, and total dry weight (Figs 2b, 3).

**Table 1:** Summary statistics and units for shoot dry weight, total dry weight, and the 24 root traits characterized in this study in winter wheat.

Trait	Abbreviation	Unit	Mean	Min	Max	*H* ^2^
Shoot dry weight	SDW	g	0.039	0.018	0.059	0.57
Total dry weight	TDW	g	0.053	0.024	0.080	0.51
Root dry weight	RDW	g	0.014	0.006	0.022	0.39
Total root respiration	TRR	nmol CO_2_ s^−1^	0.54	0.23	0.91	0.42
SRR per root length	SRR_L	nmol CO_2_ s^−1^ m^−1^	0.14	0.04	0.34	0.48
SRR per root mass	SRR_M	nmol CO_2_ s^−1^ g^−1^	39.86	23.32	74.79	0.32
SRR residual	SRR_R	nmol CO_2_ s^−1^	−0.0039	−0.3623	0.27	0.44
Specific root length	SRL	m g^−1^	299.7	182.21	398.36	0.55
Root mass fraction	RMF	%	26.48	19.05	36.17	0.43
Total root length	TRL	mm	4125.91	1315.65	7861.83	0.47
Axial root length	ARL	mm	1456.2	655.59	2537.42	0.48
Lateral root length	LRL	mm	2669.71	660.06	5494.75	0.48
Lateral : axial root length ratio	L‐to‐A_L	mm mm^−1^	1.82	0.83	2.66	0.48
Total root volume	TRV	mm^3^	329.49	135.78	610.24	0.45
Axial root volume	ARV	mm^3^	243.9	113.64	459.5	0.46
Lateral root volume	LRV	mm^3^	85.59	22.15	164.09	0.40
Lateral : axial root volume ratio	L‐to‐A_V	mm^3^ mm^−3^	0.36	0.17	0.53	0.45
Total root surface area	TSA	mm^2^	3680.62	1348.64	6477.28	0.45
Axial root surface area	ASA	mm^3^	2034.03	934.42	3639.73	0.47
Lateral root surface area	LSA	mm^3^	1646.59	414.22	3266.23	0.44
Average root diameter	AvgD	mm	0.29	0.25	0.37	0.53
Number of root tips	Tip	n	399.61	161.67	710	0.40
Number of branch points	BP	n	931.66	283	1992.5	0.54
Branching frequency	BF	n mm^−1^	0.22	0.18	0.31	0.45
Branching density	BD	n cm^−1^	2.81	1.75	5.83	0.25
Root tissue density	RTD	g cm^−3^	0.04	0.03	0.06	0.30

H^2^, broad‐sense heritability.

**Fig. 4:**
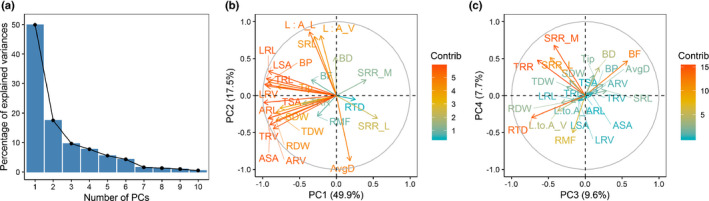
(a) Scree graph showing percentage of variance explained by each of the first 10 principal components. Principal component analysis (PCA) variable contribution plots show (b) the first and second PCs and (c) the third and fourth PCs, where relative weightings of the variables are indicated by vectors. Trait a were measured in winter wheat. ARL, axial root length; ARV, axial root volume; ASA, axial root surface area; AvgD, average root diameter; BD, branching density; BF, branching frequency; BP, number of branch points; LRL, lateral root length; LRV, lateral root volume; LSA, lateral root surface area; L : A_L, lateral : axial root length ratio; L : A_V, lateral : axial root volume ratio; RDW, root dry weight; RMF, root mass fraction; RTD, root tissue density; SDW, shoot dry weight; SRL, specific root length; SRR, specific root respiration; SRR_L, SRR per root length; SRR_M, SRR per root mass; TDW, total dry weight; Tip, number of root tips; TRL, total root length; TRR, total root respiration; TRV, total root volume; TSA, total root surface area.

Principal component analysis of the traits was conducted to further identify the major linear trait combinations that maximize the multivariate variation, and the first 10 PCs collectively explained 98.8% of the total variance. PC1, PC2, PC3 and PC4 explained 49.9%, 17.5%, 9.3% and 7.7% of the total variance, respectively (Fig. 4a). Plant size‐related traits, including TSA, TRL, TRV, TDW, RDW and SDW, made important contributions (> 5%) to PC1. By contrast, PC2 was largely driven by two structural cost related traits, AvgD and SRL, which contributed 18% and 15%, respectively (Fig. 4b). Traits with > 7% contributions to PC3 were the structural cost trait RTD (22%), three root respiration traits (TRR, SRR_L and SRR_M), and branching trait BF (14%). PC4 was predominantly driven by SRR_M and SRR_L, which represent metabolic costs and contributed 24% and 14% to the component, respectively (Fig. 4c).

### Multiple linear regression partitions respiration among root tissue types

Multiple linear regression analysis was employed to determine the respective contributions of lateral root tip, lateral root axis, and total axial root volumes to total root respiration, and to estimate the SRR_R trait. The resulting model (*P* < 2.2 × 10^–16^) explains 14.5% of the variation in total root respiration. Axial root volume, lateral root volume, and lateral root tip volume were all significant explanatory variables (*P* = 0.001, 1.37 × 10^–5^, and 0.03, respectively). The average specific root respiration rate on a volume basis of lateral root tips was 30.5 and 8.1 times the rates of axial roots and lateral roots, respectively, as determined from comparing slopes in the model (Table S1). The model provides estimates for the average SRR by volume for each root class across the diversity panel, and given the known total volume within each class for each root system, the model can predict total root system respiration. The residuals of this model are the differences between the predicted respiration and the actual respiration. Therefore, these residuals represent respiration not explained by average dependency on root type abundance within a root system, where negative values indicate that a root system respires less than expected. The residual respiration (SRR_R) ranged from −0.36 to 0.27 nmol CO_2_ s^−1^, which we hypothesized to have a genetic component.

### Trait correlation network

**Fig. 5:**
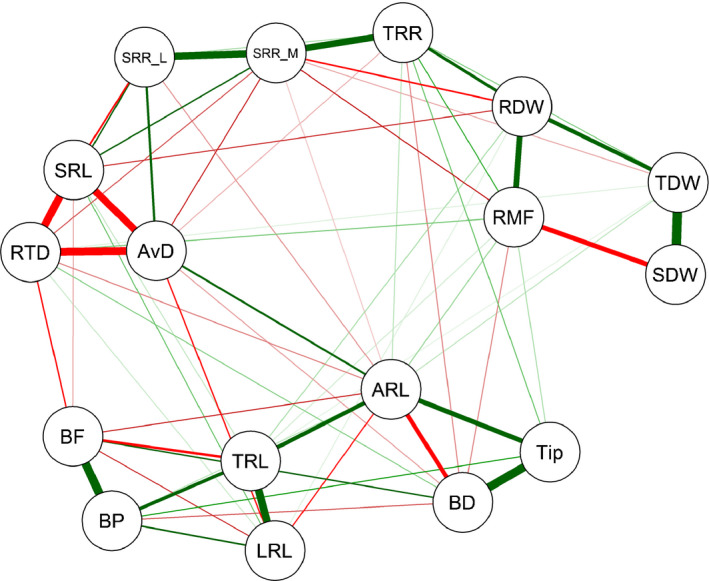
Trait correlation network constructed from the Gaussian graphical model. Red and green lines show negative and positive correlations, respectively. The cutoff was set at 0.15. Traits were measured in winter wheat. ARL, axial root length; AvgD, average root diameter; BD, branching density; BF, branching frequency; BP, number of branch points; LRL, lateral root length; RDW, root dry weight; RMF, root mass fraction; RTD, root tissue density; SDW, shoot dry weight; SRL, specific root length; SRR, specific root respiration; SRR_L, SRR per root length; SRR_M, SRR per root mass; TDW, total dry weight; Tip, number of root tips; TRL, total root length; TRR, total root respiration.

In addition to the correlation analyses, a network analysis based on a Gaussian graphical model was performed to account for the conditional dependencies between the investigated traits. The traits exhibiting an outdegree value > 2.0 were AvgD, RTD, ARL, SRR_M and SRL in descending order (Table S2). Average root diameter showed the highest betweenness, connecting a root branching subnetwork via ARL, and a biomass subnetwork via RMF. SRL also exhibited a high betweenness, by connecting other groups of traits belonging to root respiration, biomass, root morphology, and topology. Greater values for outdegree and betweenness indicate greater centrality in a network, suggesting that a trait has influence on other traits. Consistent with Pearson correlation analysis, SRR_M was weakly connected with root dry weight, total dry weight, and RMF. SRL was negatively and positively correlated with SRR_L and SRR_M, respectively (Fig. 5). In contrast to the Pearson correlation analysis (Fig. 3), no direct network connection was observed between shoot dry weight and root respiratory and architectural traits (Fig. 5).

### Genome‐wide association analysis

**Fig. 6:**
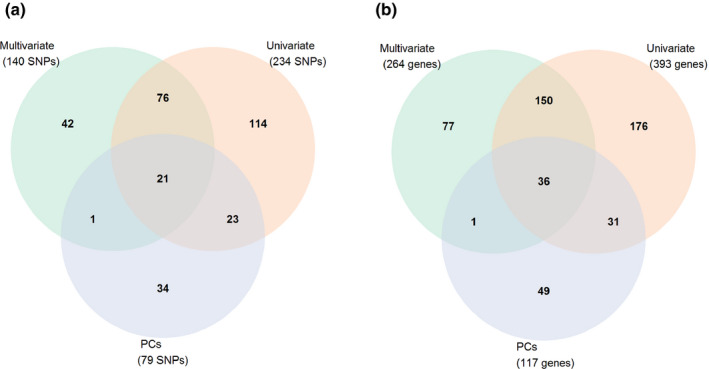
Venn diagrams of (a) associated single nucleotide polymorphisms (SNPs), with a cutoff threshold set at –log_10_P = 3.5, (b) genes identified with a cutoff set at –log_10_P = 3.5.In (a) and (b, diagrams depict the significant results for: univariate analysis of 25 traits, univariate analysis of 10 principal components (PCs), and multivariate analysis of six trait combinations.

**Fig. 7:**
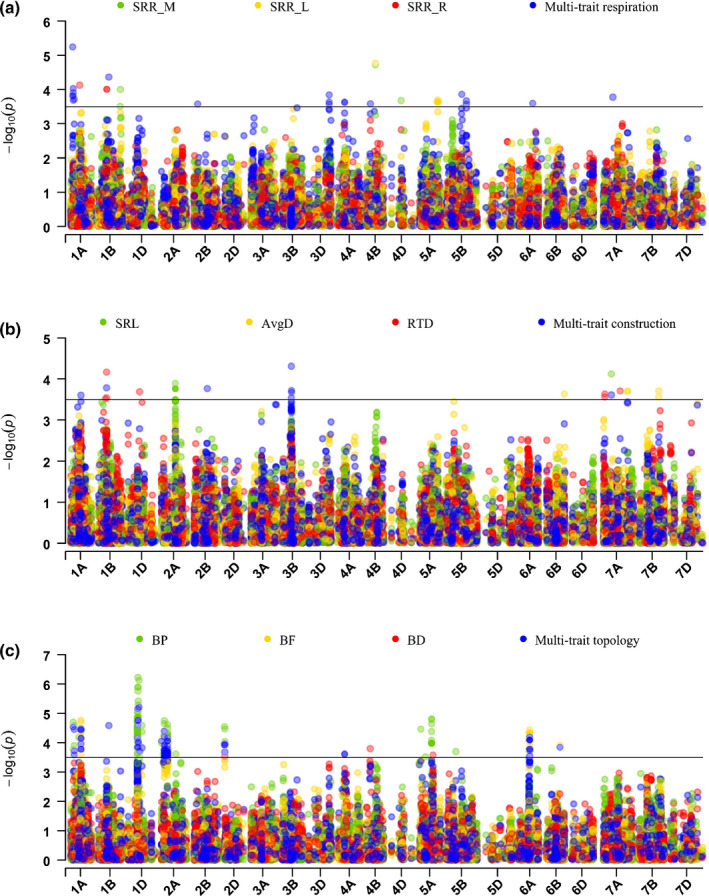
Manhattan plot of genome‐wide association study (GWAS) analyses conducted for the following traits: (a) Specific root respiration (SRR) by mass, SRR by length, residuals of total respiration vs volume of different segments, and multi‐trait combination for root respiration. (b) Specific root length (SRL), average root diameter (AvgD), root tissue density (RTD), and multi‐trait combination for root construction. (c) Number of branch points (BP), branching frequency (BF), Branching density (BD), and multi‐trait combination for root topology of the Triticeae Coordinated Agricultural Project (T‐CAP) winter wheat population. Each dot represents a single nucleotide polymorphism (SNP). The horizontal black line indicates the threshold of significance at –log_10_P = 3.5.

Multi‐trait GWAS analysis of the six sets of traits identified 140 SNPs, while the single‐trait GWAS of 25 traits identified 234 significantly associated SNPs (–log_10_ *P* > 3.5). A GWAS based on the first 10 PCs identified 79 SNPs that passed the –log_10_P of 3.5, and the majority of these detected SNPs were associated with PC1, PC2 or PC9 (Fig. 6a; Table S3). Sixty‐nine percent of the significantly associated SNPs in the multi‐trait approach and 56% of the SNPs in the PC‐GWAS were represented in the single‐trait GWAS (Fig. 6). Overall, the multi‐trait GWAS and PC‐GWAS identified 77 additional, unique SNPs that were not uncovered by the 25 univariate analyses (Figs 6a, S2). Further analysis of all identified genomic regions retrieved potential candidate genes which were within ± 250 kb of representative SNPs (Table S4), but which were much closer in general. Four significant markers associated with SRR_M were identified on chromosomes 1B, 4B and 4D (Fig. 7a). There were no candidate genes underlying the top two largest –log_10_P signals on chromosomes 1B and 4B, while the third largest –log_10_P signal (IWA430) on chromosome 4D was encoding for four potentially underlying proteolysis genes (Table S4). Seven significant markers associated with SRR_L were identified on chromosomes 4B and 5A. The marker (Excalibur_c100336_106) with the largest –log_10_P signal on chromosome 4B, which co‐associated with SRR_M, had no known potentially underlying gene. Six candidate genes near the next two largest –log_10_P signals on chromosome 5A were annotated with functions related to ATP binding, protein binding, and protein kinase activity (Table S4). Three additional significant markers associated with SRR_R were detected on chromosomes 1A and 1B (Table 2). Three candidate genes potentially underlying the largest –log_10_P signal (Kukri_c10453_875) on chromosome 1A were associated with processes of DNA transcription regulation (Table S4). There were no candidate genes found near the other two markers. The multi‐trait GWAS for root respiration identified 20 additional markers on chromosomes 1A, 1B, 2B, 3D, 4A, 4B, 5B, 6A and 7A (Fig. 7a). There were no known candidate genes underlying the largest –log_10_P signal Excalibur_c5139_198 on chromosome 1A, and four candidate genes potentially underlying the following two largest –log_10_P signals on chromosomes 1A and 1B were annotated with functions related to protein kinase activity and ADP binding (Table S4).

**Table 2:** Subset of significant single nucleotide polymorphism (SNP) markers identified from multi‐trait genome‐wide association study (GWAS) analyses in winter wheat and univariate GWAS analysis of single‐traits by selecting the top three SNPs of each trait defined in Table 1.

Trait	Model	Markers	Chr.	MAF	*P*‐value
SRR_M	Univariate	Excalibur_c100336_106	4B	0.110	1.91E‐05
SRR_M	Univariate	IAAV5776	1B	0.056	9.95E‐05
SRR_M	Univariate	IWA430	4D	0.438	2.11E‐04
SRR_L	Univariate	Excalibur_c100336_106	4B	0.110	1.70E‐05
SRR_L	Univariate	CAP12_c956_61	5A	0.112	2.04E‐04
SRR_L	Univariate	BS00066434_51	5A	0.146	2.19E‐04
SRR_R	Univariate	Kukri_c10453_875	1A	0.281	7.51E‐05
SRR_R	Univariate	IWA6965	1B	0.064	9.86E‐05
SRR_R	Univariate	RAC875_c42206_305	1B	0.064	9.86E‐05
Respiration	Multivariate	Excalibur_c5139_198	1A	0.213	5.69E‐06
Respiration	Multivariate	tplb0048b10_1365	1B	0.064	4.35E‐05
Respiration	Multivariate	Ex_c4876_1221	1A	0.248	9.36E‐05
SRL	Univariate	RAC875_c63889_486	7A	0.202	7.58E‐05
SRL	Univariate	GENE‐1220_457	2A	0.064	1.29E‐04
SRL	Univariate	RFL_Contig5917_2369	2A	0.071	1.73E‐04
AvgD	Univariate	IWA7907	7B	0.190	1.95E‐04
AvgD	Univariate	IWA4438	7A	0.083	1.97E‐04
AvgD	Univariate	Tdurum_contig61864_1352	7A	0.082	2.03E‐04
RTD	Univariate	GENE‐0249_161	1B	0.272	6.84E‐05
RTD	Univariate	IWA614	7A	0.277	1.97E‐04
RTD	Univariate	Kukri_c20062_389	1D	0.165	2.06E‐04
Construction	Multivariate	BS00082644_51	3B	0.247	4.91E‐05
Construction	Multivariate	GENE‐0249_161	1B	0.272	1.65E‐04
Construction	Multivariate	IWA6076	2B	0.273	1.72E‐04
BF	Univariate	CAP7_c1083_283	1A	0.140	1.74E‐05
BF	Univariate	Kukri_c29121_226	1A	0.140	1.98E‐05
BF	Univariate	Kukri_c53935_265	1A	0.136	3.47E‐05
BP	Univariate	IWA1464	1D	0.147	6.00E‐07
BP	Univariate	BS00032149_51	1D	0.133	7.39E‐07
BP	Univariate	IWA2164	1D	0.150	1.31E‐06
BD	Univariate	Tdurum_contig49608_1185	4B	0.143	1.59E‐04
BD	Univariate	BS00063973_51	5A	0.404	2.63E‐04
BD	Univariate	Excalibur_c33173_557	2D	0.205	2.91E‐04
Topology	Multivariate	BS00032149_51	1D	0.133	6.15E‐06
Topology	Multivariate	IWA1464	1D	0.147	6.95E‐06
Topology	Multivariate	IWA2164	1D	0.150	1.74E‐05

AvgD, average root diameter; BD, branching density; BF, branching frequency; BP, number of branch points; Chr., chromosome; MAF, minor allele frequency; RTD, root tissue density; SRL, specific root length; SRR, specific root respiration; SRR_L, SRR per root length; SRR_M, SRR per root mass; SRR_R, SRR residual.

Ten significant markers associated with single‐trait SRL were identified on chromosomes 2A (9 markers) and 7A, as well as 17 candidate genes potentially underlying the top three largest –log_10_P signals on chromosomes 2A and 7A (Fig. 7b; Table S4). Five significant markers associated with single‐trait AvgD were identified on chromosomes 6B, 7A and 7B. Only one of the top three largest –log_10_P signals on chromosome 7A had three potentially underlying genes, which were annotated with functions related to protein binding. Seven significant markers associated with single‐trait RTD were identified on chromosomes 1B, 1D and 7A, and eight candidate genes potentially underlying the top three largest –log_10_P signals on chromosomes 1B, 1D and 7A were annotated as zinc finger CW‐type coiled‐coil domain protein and integral membrane Yip1 family protein. The multi‐trait GWAS for root construction identified eight markers on chromosomes 1A, 1B, 2B, 3B and 7A, with one marker (GENE‐0249_161) on 1B co‐associated with single‐trait RTD, and another marker (RAC875_c63889_486) on 7A co‐associated with single‐trait SRL (Table 2). Eight candidate genes potentially underlying the top three largest –log_10_P signals on chromosomes 1B, 2B and 3B were annotated as regulators of VPS4 activity and potassium ion transmembrane transport (Table S4).

Significant marker associations and underlying genes were also detected for branching frequency, multi‐trait biomass, multi‐trait allocation, multi‐trait morphology, all PC‐traits except PC8, and the other single traits (Figs 6, 7c, S2, S3; Table S3).

## Discussion

Reducing the metabolic and structural carbon costs of roots has become a viable engineering strategy for crop breeding to increase yield and promote plant growth (Lynch, 2013, 2018; Amthor *et al.,* 2019). However, the genetic and functional basis of root respiration traits still lags behind architectural root traits. Scaling up phenotyping will strengthen functional phenomics of root respiration greatly by increasing statistical power and enabling genetic mapping (York, 2019). The platform we developed facilitates high‐throughput phenotyping of root respiration, with integration of cost‐effective equipment and an R script for data processing, and it allowed throughput of about 25 samples per person h^−1^. The use of a bead bath for controlling temperature avoids the risk associated with using a water bath of water entering the respiration chamber, which can result in contamination of the gas analyzer. We observed 8.5‐fold variation for SRR_L and 3.2‐fold variation for SRR_M in the wheat panel. In previous work, root respiration was measured mostly using single root segments (Poorter *et al.,* 1991; Strock *et al.,* 2018), and there was little information about how different root types impact the respiration of whole root systems. Considering the difficulty of separating different root tissue segments from whole root systems in order to maintain high throughput, multiple linear regression was used to predict the contributions of root tissue types to total root respiration of wheat seedlings on average within the panel. We found that a much higher degree of respiration was observed in the lateral root tips than in the axial root tissue or lateral root axis tissue (≤ 0.3 mm), which supports findings from studies in woody plants showing that root tip meristems consume about 15 times more O_2_ than the rest of the root system (Mancuso & Boselli, 2002; Aguilar *et al.,* 2003; Burton *et al.,* 2012).

Principal component analysis confirms a multidimensional space of root trait variation in this intraspecific wheat diversity panel. The first dimension was dominated by plant size traits which are typically not included in the trait economics literature because they are difficult to acquire for wild species, such as entire trees (Reich, 2014). Consistent with findings from seedlings of tree species by Kramer‐Walter *et al.,* (2016), a second dimension was dominated by SRL and root diameter with opposite loadings, which may reflect root adaptations for resource acquisition during breeding. SRR‐related traits loaded most strongly onto PC4, indicating that they are important drivers within the multi‐dimensional trait space measured in this panel. Intraspecific trait economics spaces have rarely been considered but may provide evidence for the underlying genetic and physiological bases of the economics space. In this work, SRL and SRR uncovered different candidate genes, which implies both can be targeted simultaneously for crop improvement because they have different developmental pathways. Reich (2014) proposed that a central feature of the trait spectra is co‐selection of the correlated single traits due to evolutionary strategies across species, which could also be true across crop varieties that are adapted to diverse environments. Crossing contrasting crop lines could be a strategy to test whether these associations can be uncoupled by genetic recombination in their progeny. If not, this may indicate that the trait associations within a multi‐trait dimension are due to inherent physical and physiological constraints rather than co‐selection as an evolutionary strategy. Consideration of trait economics spectra within crops is ripe for exploration, especially considering the advances in high‐throughput phenotyping and functional phenomics (York, 2019).

Correlation network analyses have been widely used in biology and social sciences to capture causality and precursor/product relationship patterns in functional traits. Despite the elegance of this approach, relatively few studies have applied network theory to plant root traits (Poorter *et al.,* 2014; Messier *et al.,* 2017; Carlson *et al.,* 2019; Kleyer *et al.,* 2019). In addition to root dry weight, SRL, and average diameter, SRR_M, which is rarely used in functional trait analysis, was identified as one of the hub traits, and it had substantial effects on the plant phenotype as a whole. Consistent with the findings of previous studies, SRL correlated with root dry weight, root diameter, branching, and root tissue density (Reich, 2014; Kramer‐Walter *et al.,* 2016). In addition, we found that SRL can also be an indicator of root respiration, on either a mass or length basis. Shoot biomass only had a strong positive correlation with total biomass and a negative correlation with root mass fraction in the network, which may indicate that the formation of wheat seedling shoot biomass was mostly independent, and also indicates that reducing or otherwise optimizing the allocation of resources to the root could be a strategy to improve shoot growth (Guo & York, 2019). Counterintuitively, driving shoot growth with such a strategy may actually maintain root mass and total metabolic burden, or even increase these total costs, but with a lower proportion relative to the shoot. This framework of carbon use efficiency represents an untapped positive feedback loop for plant growth. Interestingly, network, principal component, and regression analyses all showed that SRR_M was negatively correlated with total dry weight, suggesting that reducing respiratory carbon could potentially increase whole‐plant growth (Lynch, 2015; Amthor *et al.,* 2019).

Multi‐trait GWAS analysis has recently gained more attention because it often boosts SNP detection ability and assesses the full spectrum of traits that are affected by trait‐associated variants (Porter & O’Reilly, 2017), which can be particularly useful for challenging physiological traits (Chhetri *et al.,* 2019). Combining traits related to respiration, multi‐trait association analysis identified 20 unique significant associations, while the single‐trait GWAS detected 13 unique significant associations for all SRR traits. The findings potentially reveal the pleiotropic effects of genes near significantly associated SNPs on root respiration. The marker tplb0048b10_1365, the second‐largest –log_10_P signal associated with multi‐trait root respiration, was reported to be associated with nitrogen deficiency tolerance in wheat seedlings (Ren *et al.,* 2018). Multiple annotated genes potentially underlying significant SRR_L and SRR_M associated SNPs are annotated with functions in protein catabolism, protein binding, ADP, and ATP binding, which are related to cellular respiration (Araújo *et al.,* 2011), root meristem activity (Xu *et al.,* 2017) or root senescence (Liu *et al.,* 2019).

Genome‐wide association studies for root architectural traits have gained increasing attention in wheat, and several QTLs/genes in wheat have been found to associate with root architectural and morphological traits such as root length, root number, and root diameter across the genome (Maccaferri *et al.,* 2016; Ayalew *et al.,* 2018; Beyer *et al.,* 2019). Specific root length (SRL), AvgD, and RTD are important components of the root economic spectrum because they potentially provide information about root morphology and structural costs (Kramer‐Walter *et al.,* 2016; McCormack *et al.,* 2017). Multiple genes potentially underlying associated significant SNPs were identified as a zinc finger protein, a cytochrome p450 family member, and a haloacid dehalogenase‐like hydrolase family protein, all of which play important roles in controlling wheat root growth and development (Kulkarni *et al.,* 2017; Li & Wei, 2020). Multiple genes potentially underlying two markers (Kukri_c24648_262 and Kukri_c5113_1082), which were co‐associated with TRL, LRL, TRV, LRV, TSA, LSA, BP, PC1, and multi‐trait allocation and topology (Table S4), were annotated as a nucleoporin autopeptidase domain containing protein. Those genes may play distinct roles in nuclear transport and root elongation (Parry, 2014).

A recent review outlined emerging possibilities for reducing unnecessary carbon loss to increase yields (Amthor *et al.,* 2019), the findings of which were further supported by new simulation results indicating that substantial gains could be made by targeting plant respiration (Holland *et al.,* 2019). An optimal root system will therefore conform to economic cost–benefit analyses, for which the incremental cost increase associated with allocation to the root system equals the incremental benefit increase, measured as nutrient and water capture, or marginal photosynthesis (Bloom *et al.,* 1985). Recent work from the Realizing Increased Photosynthetic Efficiency (RIPE) project has also shown that it is possible to increase photosynthesis by reducing photorespiration (South *et al.,* 2019) and increasing photosynthetic induction (Acevedo‐Siaca *et al.,* 2020). This study focused on young seedlings, which is a crucial growth stage at which vigor has been shown to lead to greater yield in wheat. We propose that a combination of strategies that increase photosynthesis and decrease ‘luxury’ root respiration could have synergistic and compounding influences on plant growth. The trait economics space discussed above provides a useful framework for this strategy.

### Conclusions

We developed a high‐throughput platform for measuring multiple traits within the root economics space, including root respiration and specific root length, which are aspects of root metabolic and structural costs, respectively. Substantial, heritable variation exists within wheat, providing further evidence for intraspecific economics spectra. Employing the functional phenomics approach allowed us to leverage genetic and phenotypic diversity to infer the increased contribution of lateral root tips to respiration, the negative relationship between SRR and seedling mass, and network analysis that identified hub traits. Genome‐wide association studies for the univariate traits uncovered several underlying genetic regions, while multivariate and PCA‐based GWASs provided an improved ability to detect the genetics of the root economics space itself for the first time, to our knowledge. The SNPs associated with the traits may be useful for marker‐assisted breeding. Candidate genes underlying significant SNPs associated with root respiratory, structural, and topological traits will require further research, with the aim of reducing respiratory carbon loss and structural costs. We provide evidence that the combination of functional phenomics and trait economic theory has the potential to advance our understanding of plant biology and promote breeding of carbon use efficient crop varieties.

## Author contributions

HG and LMY conceived the research and designed the experiments. X‐FM provided the germplasm and expertise for genetic analysis. HG, AS, KD and LMY conducted experiments. HG, MG, AS, KD and LMY developed the respiration measurement protocol and R script for root respiration analysis. HG, AS, KD, HA and LMY analyzed the experimental data. HG and LMY wrote the first draft of the manuscript, all authors made revisions, and all approved the final version.

## Supporting information


**Fig. S1** Histograms showing the frequency distribution of 26 traits and 10 principal component (PC) scores.
**Fig. S2** Manhattan plots of genome‐wide association studies (GWAS) conducted on all traits.
**Fig. S3** Quantile–quantile (Q–Q) plots for all traits.
**Table S1** Multiple linear regression model describing relationships between volumes of different root segments and total root respiration.
**Table S2** Centrality measures for 17 traits from the Gaussian graphical model.
**Table S3** List of SNPs using a cutoff value set at –log_10_P = 3.5.
**Table S4** List of nearest genes underlying SNPs using a cutoff value set at –log_10_P = 3.5.
Please note: Wiley Blackwell are not responsible for the content or functionality of any Supporting Information supplied by the authors. Any queries (other than missing material) should be directed to the *New Phytologist* Central Office.

## Data Availability

All trait data, gemma output, and R analysis scripts necessary for the statistical analysis and plotting are publicly available at https://doi.org/10.5281/zenodo.4247894 (Guo *et al.,* 2020b).
